# Factors That Must Be Considered in Determining Conservative or Radical Dental Treatment

**Published:** 1920-06

**Authors:** Chalmers J. Lyons


### FACTORS THAT MUST BE CONSIDERED IN DETERMINING CONSERVATIVE OR RADICAL DENTAL TREATMENT

CHALMERS J. LYONS, D.D.SC.

One of the most important advances which has been made during the past decade in the science of medicine has been the development which disease in one part of the body plays in the etiology of disease in other parts. The recent advances in dentistry have brought this field of work into very close relationship with that of medicine. These recent advances have been stimulated by the demands of our sister profession that the dentist must assume some of the responsibility for the effects of some of the low grade infections.

The conscientious internist to-day takes into account the condition of the mouth in formulating his diagnosis in a considerable number of his medical cases. It is also desirable for the dentist to make a careful survey and inquiry into the general condition of the patient before deciding upon dental procedure. Some of his patients will require thorough and radical dental treatment, while with others a more conservative measure will be preferable.

During the past two or three decades the conservation of tooth structure has been pressed home to the public at large so forcefully that until within very recent years it was hard to find a dentist who would advise extraction of a tooth, or a patient who would submit to such an operation. During those past two or three decades, the years before the dental profession realized the importance of focal infection, when the knowledge concerning the relationship of mouth infection to systemic disease was in its infancy the dentist's main purpose was to conserve tooth structure. As a result of this extensive conservation of tooth structure many of our patients are now suffering from conditions that are far more serious than those localized in the oral cavity. As a further result of the profession's efforts in the past to conserve teeth, we are to-day confronted with a condition which threatens to destroy conservative dental treatment. To-day the pendulum is swinging out too far in the eradication of teeth for the purpose of relieving some systemic lesion.

The dentist must take a much broader view of his profession. It is a question whether dentistry as a whole even now after all that has been written on focal infection, appreciates the magnitude of its responsibilities. It is to be regretted that there are still hundreds of dentists who believe their responsibility begins and ends in conservation of tooth structure. When a patient presents himself they seem to feel that their responsibility ceases when they have examined the teeth for dental caries. They do not feel they are responsible to that patient for any other lesion that may be present in the oral cavity. They fail to examine the mucous membrane, cheeks, tongue, tonsils, sinuses, salivary glands, etc. They fail to get the patient's history of past or present illness; and they rarely take into account the resistance of the patient to disease.

It seems to the writer that the highest aim for the dentist of to-day is not merely to be a good dentist, but to be a good dental diagnostician. If he is a good diagnostician he can not help but be a good dentist.

It has been clearly demonstrated by the pathologists that many of the heart, kidney and joint lesions have their etiology in low grade infection, originating somewhere in the body. There is no question but that the subject of focal infections should be considered more seriously by both the medical and dental professions and a united front should be presented to acquire a greater knowledge of them so that they may be better controlled.

The records of the Bureau of Vital Statistics for the past twenty years are most interesting. First we see the average length of life per individual greatly increased.

In studying these mortality statistics carefully we find that the causes of deaths in some of the columns are increasing and in other columns they are decreasing. For instance there seems to be a falling off in deaths due to certain diseases such as tuberculosis, typhoid, cholera and small-pox. As we study these statistics we find an increase in the number of deaths from heart and kidney lesions and those due to arteriosclerosis and anemia.

It is interesting to review some of these statistics. In the registration area for deaths in the United States, the Vital Statistics show the death rate per 100,000 population from diseases of the circulatory system in 1901 was 161.2, while in 1917 it was 201.4; deaths from diseases of the kidneys in 1901 per 100,000 population was 97 while in 1917 it was 107.4. It is interesting in studying these statistics to observe that each year seems to show a gradual increase.

It is also interesting to observe the statistics in these diseases when there has been an effort made to get them under control. As an example the death rate from tuberculosis per 100,000 population in 1901 was 192.6, in 1917 it was 146.3. Typhoid shows a more rapid decline. In 1901 the death rate per 100,000 population was 32; in 1917 it had dropped to 13.4.

Quoting from Prof. Martin H. Fisher, of the University of Cincinnati, he says: "Many people are dying to-day from certain diseases too frequently regarded as a necessary accompaniment of old age. In my opinion," he says, "old age is not so much the cause of many of the pathologic changes attributed to it, as these changes are the causes of old age. There is a physiological death, but I question very much from the people and patients that I have seen whether a physiological death ever occurs much below 100 years. The question now comes up, if we no longer die of the old causes, and medical men concede that we do not, then what is it that carries us away in our forties, fifties and sixties? Barring gross accidents and such diseases as cancer and tuberculosis, we now die largely of the direct and indirect consequences of low grade infection.

We do not die of acute scarlet fever as we used to; we do not die of acute typhoid or acute pneumonia as we used to; and yet most of us do not die physiologically—that is, of old age. In many cities where formerly there were hundreds of cases of typhoid, to-day there are hardly enough to demonstrate the disease to medical students, but many of those who have been spared typhoid die by newer causes. These newer causes of death are subtler forms of infection and subtler types of disease. They are largely systemic infections, low grade in their origin, and spring from local causes and are so very small that they are largely overlooked.''

To-day we have hundreds of people walking about the streets, going about their business, following the pursuits of life, who are neither well nor sick, yet they complain of feeling tired all the time. They have lost their energy. They are suffering from malaise. There is a condition of restlessness, with a feeling of being uncomfortable, which is brought on undoubtedly by a certain amount of bacteria and toxines in the blood. This condition is quite common with those individuals carrying alveolar abscesses. The same symptoms may be manifest as a result of infection in any other part of the body. When these patients present themselves for treatment, all sources of low grade infection in the body must be considered and the infection eradicated wherever found. Dr. Chas. H. Mayo, in an address a short time ago, made the statement: ''As we see things to-day, it means there will be far less surgery and less sickness when the full benefit of the knowledge of low grade infections becomes known throughout the world.''

In running down these low grade infections we must not overlook the fact that multiple infection is nearly always present, and if we do not get definite and prompt response in eliminating one focus it means that we have failed to completely eradicate it, or that there are others not yet determined. In many of these cases the removal of a single focus will so lighten the patient's load that the body forces will reassert themselves and the phagocytic action of the leucocytes will take care at least temporarily of other infections. In these cases a lowering of the bodily resistance from any cause may re-establish the vicious cycle again and the patient will have a relapse. If this be true, then we are doing less than our duty towards the patient when we do not strongly recommend the removal of every possible focus of infection wherever it may be found.

These foci are not by any means easy to determine. A close co-operation between the internist and the dentist will be of great value in running down these low grade infections.

The time is coming when future generations will no longer die of disease as a result of low grade infections as we are to-day, because the source and prevention of these diseases will be known. Periods of progress in both medicine and dentistry have occurred only as scientific and general knowledge has advanced. Wonderful strides have been made in all lines of medicine during the past quarter of a century by the public's appreciation of disease and its prevention. Public health laws have been passed for the control and prevention of disease as well as for the saving of children. Such legislation has played an important role in diminishing the death rate of the nation during the last three decades.

### DENTISTRY AND PREVENTIVE MEDICINE

Are we as dentists doing all we can in the great movement for the conservation of health? The protection of human life against all destructive agencies becomes less complex when the sources and influences of these destructive agents are understood. By this knowledge Gorgas was able to free the Panama Canal Zone of disease, making it one of the most habitable places in the world. The great war was only made tolerable by prevention and control of disease and the scientific treatment of wounds.

What is the responsibility of the dental prefession to-day in the prevention and control of disease? It has been definitely demonstrated, first by Hunter of London, later by Rosenow, Billings, Hartzell, and many other American physicians and dentists that certain chronic diseases, certain acute diseases and special local diseases have their origin in a focus of infection somewhere in the human body. It has also been demonstrated that appendicitis, diseases of the gall bladder, and ulcers of the stomach are caused by a mass of bacteria in the capillary circulation at the base of the mucous cells in these organs, and is caused in the same manner from local infections originating from similar causes. It has been further demonstrated that the toxines from certain micro-organisms in the blood play a most important role in the etiology of disease.

There are several sources in the body for the entrance and culture of bacteria from local foci. It has been stated by one of our great research workers that the tonsils provide the greatest portal of entry for infection of any place in the body. Diseased teeth and alveolar tissues are unquestionably another great harbor for these micro-organisms. From the laboratory and clinical evidence that has been brought forward during the last five years there can be no question regarding the relationship between mouth infection and certain systemic lesions. The intelligent dentist to-day should have no patience with such teaching as has been brought out in a recent paper by an Eastern research worker* in which he states that he "does not believe systemic diseases upon the theory of focal infection are correctly attributable to the teeth." Quoting further, he says, "If it were true that the teeth were causative factors in the production of systemic disease, there would be few healthy individuals in the world. For example, among fifty thousand cases observed in the Forsythe Dental

---

* Howe, Dental Foci, *Dental Summary*, October, 1919.

Infirmary with all varieties of lesions involving the teeth there was not a single case of endocarditis, rheumatism, arthritis, or other systemic affection which could in any way be attributed to the teeth, that I could discover. If the theory of focal infection were sound and correct a great many of these children should have exhibited such diseases, but the fact remains that they did not.''

In the writer's opinion these statistics should be considered far from the final evidence that there is no relationship between diseased teeth and systemic disturbances. Those statistics have been compiled entirely by what has been observed *in the treatment of children's teeth.* As a matter of fact, the greatest factor to be considered in discussing this subject has entirely been overlooked by the author of those statements, and that is the normal resistance of the patient to disease. As a matter of fact, further, the normal resistance of a child is much greater than that of the adult, and they already have an established immunity to systemic lesions as a result of low grade infections. The records of our own dental clinics at the University of Michigan tell an entirely different story.

Out of eight thousand patients examined, over two thousand four hundred, or thirty per cent, gave history of some systemic lesion such as arthritis, heart or kidney involvement. In a series of cases between the ages of 25 and 40 years, where there was radiographic evidence of pyorrhea involvement or definite alveolar abscess, 58 per cent gave history of systemic lesions. It should not be understood for one moment that all of these lesions are attributable to the teeth, and this is the big question to-day that the dentist and the internist must solve. They must get these three points clearly in their minds: First, diseased dental tissues may cause systemic lesions. Second, there are many other sources of infection in the body than the teeth and dependent structures. Third, the normal resistance of the individual to disease must be the predominating factor in determining the character of the treatment.

This factor is one that has been, and is to-day, largely overlooked by the average dentist and internist. Very few adults are without some form of infection in some part of the mouth, and the knowledge of the same is not known to the patient. That serious secondary manifestations are not more common as a result of these foci is probably due to two factors: First, the normal resistance of the individual is such that bacterial invasion into the lymph and blood streams is repelled. Second, the bacteria of chronic processes are usually of relative low virulence. If it were probable that the individual's resistance to disease would remain high, and that the bacteria in these foci would remain of low virulence, then we would not need to be alarmed over the problems of focal infection. However, Rosenow has shown that these pathogenic organisms may not always remain of low virulence. He believes they have the property of mutation, whereby they may be of low virulence to-day when the individual's resistance is sufficiently high to take care of them. Later, if the individual's resistance becomes lowered, the future generations of the same low virulent pathogenic micro-organisms may take on different characteristics and with suitable environment and nutrition may become exceedingly virulent.

On the part of the individual, the resistance to infection may be lowered by a large variety of causes, such as drug habits, tuberculosis, anemia and other blood diseases, diseases of the ductless glands, mineral poisons, pregnancy, old age, overwork, lack of proper nourishment, faulty methods of living, exposures to cold, etc., etc. In these patients oral and other forms of sepsis are a greater menace to health than is the case with normal individuals.

Dentistry to-day must meet and encounter these problems squarely if it is to take the place it deserves in the conservation of the public health. Dentistry can no longer be practised as it has been in the past. In the years gone by the profession has been chiefly engaged in the conservation of tooth structure without reference to or knowledge of the fact that in such practice they were subjecting many of their patients to serious systemic disturbances. To-day the knowledge of the relationship of mouth infection to systemic disease is becoming known throughout the world, and there should be no excuse for the dentist to continue the practice of tooth conservation as he has done in the past.

In the writer's opinion we are not yet ready unconditionally to accept the teaching of some that all pulpless teeth are a menace to humanity and should be extracted. Such teaching should be condemned as unscientific. We are not ready either to accept the teaching that all classes of patients may tolerate pulpless teeth. There surely must be a sane ground for dentistry to take which will lead to a basis for a safer practice.

The dentist who will build and mold a practice for the future which will be safe for his patients, will practice conservative treatment or radical dentistry. The determination of the future dental treatments must be based more and more upon the results obtained by a thorough diagnosis. The dentist must assume a greater responsibility to his patient than the mere restoration of the masticatory organs.

The resistance of the patient to disease must be uppermost in the dentist's mind when treating pulpless teeth. We must not overlook the fact that certain types of patients may tolerate successfully a certain class of dental work, which would result in a failure for another patient of less resistance. If the patient's resistance is low, radical dentistry should be indicated. In the writer's opinion the resistance of the patient to disease must be the great determining factor in the practice of conservative or radical dentistry. There never was a time in the history of dentistry when a close co-operation between the physician and the dentist was as necessary as now. There was never a time in medicine when the physician needed the advice of a dentist as at the present time.

To-day the problem of focal infection is one that must be studied by both professions, and can only be solved by absolute harmony and the closest co-operation between the two. It is unfortunate to-day that both the physician and the dentist are placing so much confidence and dependence upon X-ray findings in formulating their diagnosis. They seem to overlook the fact that the radiogram does not show a picture of the pathology, but it only shows shadows of conditions which may be present in the tissues. Many conditions may be read into the radiogram by the inexperienced. In the writer's opinion the radiogram *per se* is of little value in making a judicial diagnosis. The radiogram may be misleading many times if there is no other evidence at hand. It may show rarefied areas about the jaws that are physiologic rather than pathologic. It may also show rarefied areas that are traumatic and not infectious. It should be used only in conjunction with the case history, physical diagnosis, etc. It should be considered only one link in the whole chain of evidence from which may be formulated a sane and conservative diagnosis.

Our knowledge of the far-reaching effects of low grade infection should be sufficient reason for eradicating a focus of infection wherever it may be found. Too often teeth are condemned by the physician when the origin of the infection may be in some other part of the body.

A famous internist* recently made this statement: "There is no doubt that many cases of arthritis and myositis have been relieved and many eyes saved, and the general health of many patients improved through the reference to dentists to have corrected bad mouth conditions. One error for which the physicians are mainly at fault, is that arising from a tendency to make incomplete examinations. A patient complains of arthritis or other

---

* Dr. E. E. Irons, Chicago, *Jour. Nat. Dent. Assn.*, November, 1919.

pain, his physician looks into his mouth, sees a few crowns, and at once condemns the teeth and advises their sacrifice, and the conscientious dentist has great difficulty in convincing the patient that the crowns are on sound roots. Now the fact is in a case of this sort the physician may be right, but, taking all things into consideration, he is more likely to be wrong. What the physician should do is to give the patient a very careful and complete examination, with the co-operation of the dentist, and then decide upon the course of action. Some years ago many alveolar abscesses remained undiscovered and undisturbed. When the dental and medical professions discovered their omission they went to the opposite extremes. Undoubtedly alveolar abscesses should be relieved, but expert dental judgment is required before condemning teeth on imperfect radiographic evidence."

There will be many cases where the patient's welfare will be better safeguarded if the dentist co-operates with the physician relative to the physical condition of the patient before he determines upon conservative or radical treatment. Too often the dentist is over-conservative in the treatment of teeth, and will attempt to retain a pulpless tooth in a patient's mouth whose physical resistance will not tolerate it. No attempt should be made to save a tooth, if in so doing it destroys the health of the patient. The writer here wishes to take exception to the statement made by some physicians and a few dentists that a pulpless tooth acts as a foreign body in the jaw.

Many men in the profession believe that if the pericemental membrane is vital and if the canals have been sterilized and properly filled that the tooth in question may be retained for many years of usefulness, providing the resistance of the patient is normal.

The foundation upon which the treatment of pulpless teeth must stand is based upon three factors: first, the amount of pathologic involvement; second, the physical condition of the patient; third, the possibility of sterilizing and completely filling the root canals.

If all the evidence shows that the end of the root is not denuded of its pericemental membrane the case can be watched and a series of radiograms be taken at stated intervals and compared. The patient should be apprised of the fact that this particular tooth is under suspicion. Here again the general health of the patient must be considered and the technic of sterilizing and filling the root canals must be considered part of the chain of evidence which must govern our procedure.

If, however, a case presents an alveolar abscess of long standing or a case of imperfectly filled root canals with granuloma, where all the evidence points to a non-vital pericemental membrane in the apical area, surgical procedure rather than dental therapy is indicated. At the present time there is no method or medicament that will revivify the pericemental apex. If this membrane be vital, the tooth is healthy; if it be diseased that portion should be eradicated. This is the point in treatment where materia medica should stop and where good surgery should begin.

The character of the surgical procedure may be root resection in favorable teeth and extraction of the tooth in unfavorable cases.

Again we approach the subject of diagnosis. What are to be considered favorable cases for root resection? It is a lamentable fact that many men are resorting to this operation as a short-cut method of eradicating an alveolar abscess over a beautiful crown or bridge attachment without first removing the crown or bridgework and sterilizing and properly filling the root canals.

Thoma in his book on "Oral Abscesses" says, "Apicoectomy is only successful if the root canal has been sterilized and properly filled previous to the operation. It is not sufficient simply to amputate the root where the old filling ends, but the root canals and dentinal tubules have to be sterilized or there will be reinfection from the tubules exposed where the root is cut. If it is not worth while to remove a crown and treat the root canal, the tooth should be extracted or there will be a recurrence and the patient is as badly off as before. It is not justifiable to leave a crown on a tooth because it is a masterpiece of art, if the foundation upon which it is built ruins the patient's health.'' This expresses the writer's views exactly. Too many times this operation is done with the view of saving a nice piece of bridgework when no attempt is made to eradicate the source of the infection. It should be a matter of routine not to do this operation unless the canals and tubuli have been sterilized and filled just previous to the operation. We are not doing good surgery when this is not done.

What are the most favorable teeth for the operation? For some time the writer has been limiting this operation to single-rooted teeth. This limitation has been made for two reasons: First, because of the accessibility of these teeth, a clean surgical operation can be performed. Second, we can be more sure of our sterilization and filling of the root canals. The possibilities of reduced silver* in the preparation of roots for resections seem to be encouraging.

The silver solution used for rapid reduction is made up as follows: Silver oxide is precipitated from a silver nitrate solution with KOH. This is carefully washed to remove all impurities and kept moist in a small amber-colored bottle. In this condition reduction is so slight that it has been kept for a long time without much change. If a small amount is insoluble in excess of ammonia, there has been too much reduction and the silver oxide should be freshly prepared. This is the stock solution and it will be seen that it is more desirable than the ammoniacal solution made from silver nitrate, because it is free from nitric acid and other impurities.

---

* This is a modification of the Howe method for silver reduction advocated by Dr. W. G. Rickert, *Nat. Dental Jr.*, October, 1919.

Now when we desire rapid reduction as for apicoectomy, the silver oxide is added to a drop or two of ammonium hydroxide to the point of saturation. This forms silver ammonium oxide. In this state the ammoniacal solution is easily reduced. The ease with which reduction takes place with this solution is so marked that even burnishing with a warm glass rod is sufficient to reduce to the lustrous metallic silver. There is one precaution that must be mentioned here. That is that after a few hours, fulminates of silver may be formed from the ammoniacal solutions, which are very explosive. Serious accidents are possible from even wet solutions. Only very small amounts of ammoniacal solutions should be made and the unused portion discarded immediately after treatment. After complete reduction there is no danger and only old ammoniacal solutions, especially after drying, are to be avoided.

The following technic is the one used preparatory to resection: The canals are opened, using the rubber dam, and the silver solution is introduced to the very apex. Five or ten minutes are then allowed for penetration before the reducing agent is applied. This treatment is applied several times. The canals are then very carefully filled by packing with small pieces of gutta-percha points softened by gently heating.

The question of sterilizing and properly filling canals in multi-rooted teeth for the eradication of an infection has been a very doubtful procedure in the mind of the writer and in his opinion the patient's welfare will be better taken care of in these cases by extraction of the tooth followed by curettage of the bone.

It is possible in a few cases to resect the entire lingual root of an upper molar or the mesial or distal root of a lower molar and preserve the remainder of the tooth for further usefulness for a limited time. Such procedure, however, is usually only temporizing and if the patient is suffering from systemic disturbance the operation would under no circumstances be indicated.

Where the bone and pericemental membrane are diseased from the apex to the gingival margin of the gums the tooth should be extracted rather than try to save it by root resection. In no case should this operation be resorted to when the tissues are diseased beyond the apical third of the root of the tooth.

If this operation is to take its place in conservative dental practice, our diagnosis will play a very important role. Our whole success will depend upon whether our diagnosis has been correct. The modus operandi will avail nothing towards success of the operation if our diagnosis has been faulty,

The welfare of the patient should be the first consideration and a hasty diagnosis may lead to just simply an operation rather than a judicious and successful treatment of the condition.

There are many men in both dental and medical professions that are not yet aware of the significance of low grade infections. A closer co-operation between medicine and dentistry will avail much in accomplishing the desired results—yet both doctor and dentist must be awake to the true situation of affairs. It will not avail the physician anything if he refers his patients to a dentist who is not awake to this serious situation. He should know who is who in dentistry and many times if he expects to get the desired results it will not be possible for him to co-operate with the patient's own dentist unless that dentist knows the situation. The same is true with the dentist. In many of these cases the patient's welfare will be better safeguarded if the dentist co-operates with the physician. He should know who is who in medicine, and if the patient's family physician does not fully recognize the importance of these conditions, then he is not doing the best that might be done for the patient in referring the patient to him for co-operation. This may sometimes result in an embarrassing situation, yet the time is here when both the dental and medical profession must be awakened to the serious situation of these low grade infections. The time is here when the patient will not be referred to a dentist with the orders for a complete removal of all of the teeth, but will be referred to the dentist for advice and co-operation. When the time comes that the doctor and dentist are working together for the best interest of their patients, then will be the time that both professions will justly receive the blessings of a grateful humanity.—*Oral Health,* April, 1920.

